# EXPLORING THE RELATIONSHIP AMONG PLACE ATTACHMENT, SELF-PERCEPTION OF AGING, AND SOCIAL PARTICIPATION

**DOI:** 10.1093/geroni/igad104.3336

**Published:** 2023-12-21

**Authors:** Wei-An Chen, Li-Fan Liu

**Affiliations:** National Cheng Kung University, Tainan, New Taipei, Taiwan (Republic of China); National Cheng Kung University, Tainan, New Taipei, Taiwan (Republic of China)

## Abstract

In the context of aging in place, the purpose of the study was to explore the relationship between place attachment, self-perception of aging and social participation of middle-aged and older adults living in the community. The study sample consisted of 226 people aged 50 and over that lived in an urban community in Tainan city, Taiwan. The instrument used were questionnaires that included socio-demographics, health status, and scales for the three main variables. The data was analyzed using correlation, linear regression and mediation testing. The result showed a significant positive correlation between place attachment and both positive and negative self-perception of aging. Furthermore, it was found that negative self-perception of aging and place attachment had significant effects on social participation. Specifically, individuals with lower levels of negative self-perception of aging and higher levels of place attachment showed higher levels of social participation. Finally, the mediation analysis indicated that place attachment played a partial mediating role in the relationship between negative self-perception of aging and social participation. This means that under the positive effect of place attachment, social participation of middle-aged and older adults with negative self-perception of aging can still be increased. In conclusion, based on the interaction between aging and the environment, in addition to the importance of reducing negative views on aging, this study sheds light on the power of emotional bonds with living place in promoting active and healthy aging in familiar neighborhoods.

